# Systemic perturbations in amino acids/amino acid derivatives and tryptophan pathway metabolites associated with murine influenza A virus infection

**DOI:** 10.1186/s12985-023-02239-0

**Published:** 2023-11-21

**Authors:** Huda A. M. Al-Shalan, Lu Zhou, Zhifan Dong, Penghao Wang, Philip K. Nicholls, Berin Boughton, Philip A. Stumbles, Wayne K. Greene, Bin Ma

**Affiliations:** 1 School of Medical, Molecular and Forensic Sciences, Murdoch University, Murdoch, WA Australia; 2 Department of Microbiology/Virology, College of Veterinary Medicine, Baghdad University, Baghdad, Iraq; 3 Graduate School, Hebei Medical University, Shijiazhuang, Hebei China; 4 Australian National Phenome Centre, Computational and Systems Medicine, Health Futures Institute, Murdoch University, Murdoch, WA Australia; 5 Telethon Kids Institute, Perth Children’s Hospital, Nedlands, WA Australia

**Keywords:** Influenza A virus, Influenza, Amino acids, Tryptophan pathway, Infection, Metabolites, Immunometabolism, Neurotoxicity

## Abstract

**Background:**

Influenza A virus (IAV) is the only influenza virus causing flu pandemics (i.e., global epidemics of flu disease). Influenza (the flu) is a highly contagious disease that can be deadly, especially in high-risk groups. Worldwide, these annual epidemics are estimated to result in about 3 to 5 million cases of severe illness and in about 290,000 to 650,000 respiratory deaths. We intend to reveal the effect of IAV infection on the host′s metabolism, immune response, and neurotoxicity by using a mouse IAV infection model.

**Methods:**

51 metabolites of murine blood plasma (33 amino acids/amino acid derivatives (AADs) and 18 metabolites of the tryptophan pathway) were analyzed by using Ultra-High-Performance Liquid Chromatography-Mass Spectrometry with Electrospray Ionization at the acute (7 days post-infection (dpi)), resolution (14 dpi), and recovery (21 dpi) stages of the virus infection in comparison with controls.

**Results:**

Among the 33 biogenic amino acids/AADs, the levels of five amino acids/AADs (1-methylhistidine, 5-oxoproline, α-aminobutyric acid, glutamine, and taurine) increased by 7 dpi, whereas the levels of ten amino acids/AADs (4-hydroxyproline, alanine, arginine, asparagine, cysteine, citrulline, glycine, methionine, proline, and tyrosine) decreased. By 14 dpi, the levels of one AAD (3-methylhistidine) increased, whereas the levels of five amino acids/AADs (α-aminobutyric acid, aminoadipic acid, methionine, threonine, valine) decreased. Among the 18 metabolites from the tryptophan pathway, the levels of kynurenine, quinolinic acid, hydroxykynurenine increased by 7 dpi, whereas the levels of indole-3-acetic acid and nicotinamide riboside decreased.

**Conclusions:**

Our data may facilitate understanding the molecular mechanisms of host responses to IAV infection and provide a basis for discovering potential new mechanistic, diagnostic, and prognostic biomarkers and therapeutic targets for IAV infection.

**Supplementary Information:**

The online version contains supplementary material available at 10.1186/s12985-023-02239-0.

## Background

Each year 5% to 10% of adults and 20% to 30% of children suffer from influenza worldwide [1]. Four types of influenza viruses are known: A, B, C, and D. Influenza A virus (IAV) and Influenza B virus can cause seasonal epidemics of disease (known as the flu season) almost every winter in many countries. However, IAVs are the only influenza viruses causing flu pandemics (i.e., global epidemics of flu disease) [1–3]. As a single-stranded RNA virus, IAV is most likely to mutate, and influenza pandemics are typically caused by the emergence of new subtypes (or the reappearance of old subtypes) of IAVs [2, 3].

Like severe acute respiratory syndrome coronavirus 2 (SARS-CoV-2), Influenza viruses are most commonly spread by the inhalation of infectious respiratory droplets produced by an infected person while talking, coughing, or sneezing [3]. Influenza can also be spread through the touching of surfaces on which infectious droplets have landed [3]. The clinical spectrum of seasonal flu can range from asymptomatic infection, uncomplicated upper-respiratory-tract symptoms (with/without fever), to severe complications (e.g., severe pneumonia, severe heart/ kidney disease, and multiple organ failures leading to death) [3]. Worldwide, according to the World Health Organization, these annual epidemics are estimated to result in about 3 to 5 million cases of severe illness and in about 290,000 to 650,000 respiratory deaths. In addition, co-infection of SARS-CoV-2 with other respiratory pathogens (e.g., IAV), which may complicate the diagnosis, treatment, and prognosis of coronavirus disease of 2019 (COVID-19), has emerged as a new concern globally. COVID-19 and influenza, as two epidemics at the same time, can occur in the cold months of the year [4]. Therefore, Influenza A is a global health concern and can also cause massive economic loss because of inpatient/outpatient care settings and substantial indirect costs attributable to lost productivity [1].

Upon detection of IAV, the host immune system is activated to defend against and clear the viral infection. The innate immune system, which provides the first line of defense against IAV infection, consists of physical/anatomical barriers and effector cells (e.g., macrophages, neutrophils, and natural killer cells), antimicrobial peptides, soluble mediators (e.g., tumor necrosis factor α (TNF-α), interferon‐γ (IFN-γ)), seric proteins (e.g., complement system and collectins), and cell receptors (e.g., Toll-like receptors and RIG-I-like receptors (sensing viral RNA)) [5, 6]. The adaptive immune system comprises two main mechanisms: humoral immunity and cellular immunity. The humoral immune response acts through hemagglutinin (HA)-specific circulating antibodies to neutralize the IAVs. Cellular immunity mediated by T lymphocytes also plays a key role in fighting against IAV, despite the presence of neutralizing antibodies [5, 6]. Indeed, to evade host immune surveillance, IAVs have developed multiple strategies for successful invasion and replication [5, 6].

The term "metabolism" refers to all the chemical processes that allow life and the normal functioning of human/animal bodies. Its three main functions are (1) the conversion of the energy in nutrients into energy for cellular activities/processes, (2) the conversion of nutrients into the building blocks of proteins (e.g., amino acids), lipids (e.g., glycerol and fatty acids), nucleic acids (e.g., nucleotide), and some carbohydrates (e.g., glucose, galactose, and fructose), and (3) the elimination of metabolic waste products (e.g., ammonia, urea, uric acid, and creatinine) [7]. All these biochemical processes have been closely linked with immunity. For example, recent studies have shown that both IAV infection and host responses alter host metabolism [8, 9]. In addition, IAV infection is a potentially life-threatening disease, especially in individuals whose immune system is compromised or in individuals with chronic medical conditions such as metabolic disorders (e.g., diabetes) [10, 11]. Cellular metabolism can also affect the immune cell state/fate and contributes to infectious diseases, inflammation, cancers, and other diseases [12, 13]. Furthermore, interaction/crosstalk between the immune and metabolic systems can mediate homeostasis in the human body [12, 13]. Therefore, research into the pathophysiological/metabolic mechanisms involved in IAV infection might facilitate improvements in the diagnosis, treatment, and control of multiple viral diseases [14].

The changes in the concentrations of amino acids/amino acid derivatives (AADs) following a virus infection can vary depending on the specific virus, the host organism, the stage of infection, and other factors [15]. Infections can trigger complex immune responses with possible effects on amino acid metabolism. First, the demand for certain amino acids increases [16]. For example, arginine, glutamine, and cysteine are known to be crucial for immune responses. Second, protein synthesis is altered during the infection [17]. Third, in response to infection, the body often initiates an inflammatory response that can influence amino acid metabolism and availability [15]. Fourth, amino acids (e.g., those amino acids that are neurotransmitters) can act as signaling molecules that modulate immune responses [18]. Their concentrations might change to regulate the immune system during an infection. Last, viral infections, especially gastrointestinal viruses, can alter the composition of the gut microbiota, which can, in turn, influence amino acid metabolism [19].

Tryptophan is an essential amino acid containing an α-amino group, an α-carboxylic acid group, and a side chain indole [20]. The metabolism of tryptophan occurs via the kynurenine pathway or the serotonin pathway to produce bioactive metabolites. Tryptophan plays a unique role in metabolic immune regulation, whereby the immune response is modulated by tryptophan and other associated metabolites or the nutrient supply. Numerous studies have linked tryptophan and its metabolites/pathways to viral infections such as COVID-19 [15, 21–23]. For example, tryptophan and its metabolites (including melatonin) might improve the host immune response and reduce inflammation during COVID-19 infections [24]. In addition, the tryptophan pathway is associated with depression, musculoskeletal pathology, and pathophysiological conditions in COVID-19 [22, 25, 26].

Metabolomics is an emerging discipline that involves the qualitative and quantitative analysis of all small-molecular-weight metabolites of a particular cell, tissue/organ, or organism [27]. The results obtained directly reflect the current state of the biological system and any changes in the overall metabolites related to the specific pathophysiological state of the body, thereby providing a new research tool for the diagnosis and treatment of diseases [15, 27, 28]. For example, metabolomic analysis has been extensively utilized for the study of the pathophysiology, diagnosis, treatment, and prognosis of COVID-19 [12, 15, 29]. In addition, a few studies have also been performed on the pathophysiology, diagnosis, treatment, and prognosis of IAV infection in vitro and in vivo [8, 9, 30]. However, a need remains for further understanding the systemic role of altered metabolic states in the host response to IAV infection, through comprehension of the crosstalk of the immune system and metabolic system.

Since our particular interest is the neuroimmune crosstalk/interaction that occurs in health and diseases [31–33], we wish to focus on the metabolites related not only to the immune response/inflammation, but also to the neurological function, pathology, and toxicity that are found during infection. Because amino acids are precursors of various biomolecules (e.g., neurotransmitters/neuropeptides and cytokines) that can impact neuronal/immune functions, and because their levels are altered in infection/inflammation [15, 34, 35] and neurological/neuroimmunological diseases/disorders [36, 37], our aim has been to analyze systemic amino acid-related (biogenic and tryptophan pathway) metabolic changes after IAV infection by using a mouse-adapted human IAV infection model.

## Methods

All animal experiments were performed according to the recommendations of the National Health and Medical Research Council of Australia in Guidelines to Promote the Wellbeing of Animals Used for Scientific Research. All experimental procedures were approved by the Animal Experimental Ethics Committee of the Harry Perkins Institute of Medical Research (Permit number: AE189).

### Virus

Mouse-adapted human IAV H1N1 PR8 (Influenza A/Puerto Rico/8/1934, H1N1; from the American Type Tissue Culture Collection) was prepared as previously described [38]. Briefly, the H1N1 virus was prepared from allantoic fluid of 10-day-old embryonated chicken eggs. The stock virus was sub-passaged through Madin-Darby canine kidney (MDCK) cells in Dulbecco´s modified Eagle′s medium (DMEM; Gibco, Sydney, Australia) and harvested as tissue culture supernatant. The viral titers were determined by cytopathic effects on MDCK cells and expressed as the mean log 10 tissue culture infective dose that killed 50% of the cells (TCID 50) over a 5-day incubation period [38]. The cytopathic effect was examined by light microscopy (without staining).

### Mouse infection and blood sample collection

Seven-week-old female C57BL/J6 mice were purchased from the Animal Resource Centre (Perth, WA, Australia). All the mice were housed in individually ventilated filter cages with autoclaved food under specific pathogen-free conditions and a 12-h dark/light cycle (room temperature: 25 °C; relative humidity: 60%). They were randomly divided into six groups: three control groups and three infected groups, with each group consisting of 5 mice. The mice were inoculated intranasally (i.n.) under light inhalation anesthesia (with isoflurane (Sigma)) with 20 TCID 50 H1N1 diluted in 50 µl sterile phosphate-buffered saline (Sigma).

Mice were monitored daily by checking their weight and potential signs and symptoms of diseases during the 21-day experiment. Animals were humanely killed in accordance with the approved animal protocol if they lost ≥ 25% of their original body weight at various time points (or at the indicated time points) and were scored as dead and/or displaying severe clinical features of the disease (e.g., lethargy, ruffled fur, labored breathing, hunched posture, and huddling behavior).

Blood was taken by a cardiac puncture technique (by using a 1 ml syringe with a 25G needle) from the mice at 7-, 14-, and 21-days post-infection (dpi) under anesthesia. Each blood sample was transferred into a MiniCollect® TUBE 1 ml K3E K3EDTA lavender cap (Greiner Bio-one, Kremsmünster, Austria) and kept at 4 °C for 24 h. It was then centrifuged at 1500 × g for 10 min in a refrigerated centrifuge. The resulting supernatant was designated as plasma. The plasma sample was kept at − 80 °C until analysis.

### Quantification of metabolic phenotyping panel

Although heat treatment (56 °C for 30 min) has previously been used in viral inactivation [39], our samples were not heat-treated before analysis because heat treatment might cause significant changes in the metabolite profiles of some complex pathways. Ultra-High-Performance Liquid Chromatography-Mass Spectrometry (UHPLC-MS) was used to measure the concentrations of 51 metabolites in the plasma samples obtained from infected and uninfected mice at 7, 14, and 21 dpi. The employed protocol included standard/validated assays and produced a complete list and the quantification of amino acids/AADs and metabolites of the tryptophan pathway [15].

### Biogenic amino acids/AADs analysis

Thirty-three amino acids/AADs were measured as previously described [15, 40]. They were quantified from 10 μl plasma samples. The extraction of plasma samples was completed using a Biomek i5 sample automation system (Beckman Coulter, Mount Waverley, Australia).

After the plasma samples had been diluted 1:1 with water, 20 μl water-stable isotope-labeled (SIL) internal standards (12.5 µmol/L generated from Canonical/Non-canonical Amino-acid mix sets (MSK-CNCAA), Cambridge Isotope Laboratories, Tewksbury, MA, USA) were added, and protein precipitation was achieved by the addition of 90 μl methanol (Sigma) followed by mixing and centrifugation. 10 μl of the resulting supernatant was transferred into a Waters 700 μl 96-well plate for derivatization using borate buffer (70 µl) and AccQTag Ultra reagent (20 µl, Waters Corp., Milford, USA) [40, 41]. The samples were mixed and incubated at 55 °C for 10 min. Subsequently, the derivatized samples were diluted 1:4 (v/v) with water before being analyzed with a Liquid Chromatography-Mass Spectrometry (LC–MS) instrument. For the LC–MS analysis, we used a Waters Acquity I-class UHPLC System (comprising a Binary Solvent Manager, thermostatic Column Manager and FL Sample Manager, Waters Corp., Milford, MA, USA) using an Acquity UPLC HSS T3 1.8 µm 2.1 S-4 × 150 mm column. Eluent A consisted of 2 mM ammonium acetate in water and eluent B consisted of 2 mM ammonium acetate acetonitrile/water 95/5 (v/v). The flow rate was 0.6 mL/min and column temperature was maintained at 45 °C. The autosampler compartment was cooled to 4 °C and 2 µL injection volume was performed using full-loop injection mode. Gradient elution was performed starting with 5% B for 0.2 min, increasing to 30% B at 5 min, 100% B at 5.1 min for 1 min before returning to 5% B until 7.5 min. The weak and strong washes were water/acetonitrile 95/5 (v/v) and isopropanol, respectively. Positive electrospray ionization (ESI) was performed on a quadrupole time-of-flight (QToF) mass spectrometer (Bruker Impact II (Bruker Daltonics, Billerica, MA)) [40] operated in broadband collision-induced dissociation (bbCID) mode. This bbCID function offers MS and Tandem Mass Spectrometry (MS/MS) spectra within the same injection. The ion source settings were: capillary voltage = 4.5 kV; end plate offset = 500 V; drying gas flow = 12.0 L/min; nebulizer gas = 5.0 bar; drying temperature = 250 °C. The data acquisition rate was set to 8 Hz over the mass range of m/z 30 – 1000. The collision energy for the MS scan was set to 6.0 eV and alternating low and high energy for MS/MS were set at 20 and 50 eV. An internal calibration was performed by injection of 5 mM sodium formate solution in water:isopropanol (50:50 v/v) at the beginning of every run.

The obtained raw data files were processed for peak integrations and for the calculation of metabolite concentrations by using Target Analysis for Screening and Quantification (TASQ) software v2.2 (Bruker Daltonics, Bremen, Germany) [15].

### Analysis of tryptophan metabolic pathway

We analyzed 18 metabolites of the tryptophan metabolic pathway as previously described [15]. Tryptophan metabolites were extracted from 50 μl plasma samples by means of a Biomek i5 sample automation system. SIL internal standards (20 μl, Novachem, VIC, Australia) were added to all the samples prior to protein precipitation (by adding 250 μl methanol containing 2 mM ammonium formate).

After being mixed, the samples were transferred into a Phenomenex PHREE phospholipid removal solid-phase extraction plate (Phenomenex, NSW, Australia). The PHREE plates were then washed with 150 μl methanol containing 2 mM ammonium formate. The eluent collection plates were dried in a SpeedVac vacuum concentrator (Thermo Fisher, Waltham, MA, USA). Before being analyzed by LC–MS, the dried extracts were resuspended in 100 μl water containing 0.1% formic acid (Sigma).

LC–MS analysis was performed using a Waters Acquity UPLC® (Waters Corp., Milford, MA, USA) coupled to a Waters Xevo TQ-XS MS (Waters Corp., Wilmslow, UK). The LC column used S-6 was a Waters HSS T3 2.1 × 150 mm, 1.8 μm column maintained at 45 °C. Linear gradient elution was performed at 0.6 mL/min. The mobile phase was composed of 0.1% formic acid in 2 mM ammonium formate (v/v) (A) and 0.1% formic acid in acetonitrile (v/v) (B), starting at 1% B increasing to 10% B over 3 min, then increasing to 90% B at 4 min, and finally returning to 1% B at 4.1 min for column re-equilibration, which was completed at 5 min. The weak and the strong washes were 95:5 water/acetonitrile (0.2% formic acid) (v/v) and 100% isopropanol (0.5% formic acid), respectively.

### Statistical analysis

Supervised multivariate statistical modeling was performed with the combined set of 51 analytes (amino acids/AAD and tryptophan catabolic metabolites). Data were log-transformed and autoscaled prior to modeling. We used mean (average) concentrations of plasma samples from 5 mice for comparison. Statistically significant difference was evaluated using unpaired Student′s t-test. Differences were considered statistically significant if the p-value was smaller than 0.05.

## Results

### Amino acid/AAD profiles of IAV-infected mice

Mice were infected with IAV, and blood was withdrawn at the acute/peak (7 dpi), late/resolution (14 dpi), and recovery (21 dpi) stages for the analysis of 33 amino acids/AADs in blood plasma. During the experiment, all animals survived without having any of the critical conditions that would have necessitated their being humanely euthanized. After PR8 infection, animals quickly lost body weight at 2–7 dpi and then gradually recovered reaching their starting weights at 14 dpi. The changes of body weight at 3 dpi, 7 dpi, 14 dpi, and 21 dpi are shown in Additional file 1: Fig. S1.

**Fig. 1:**
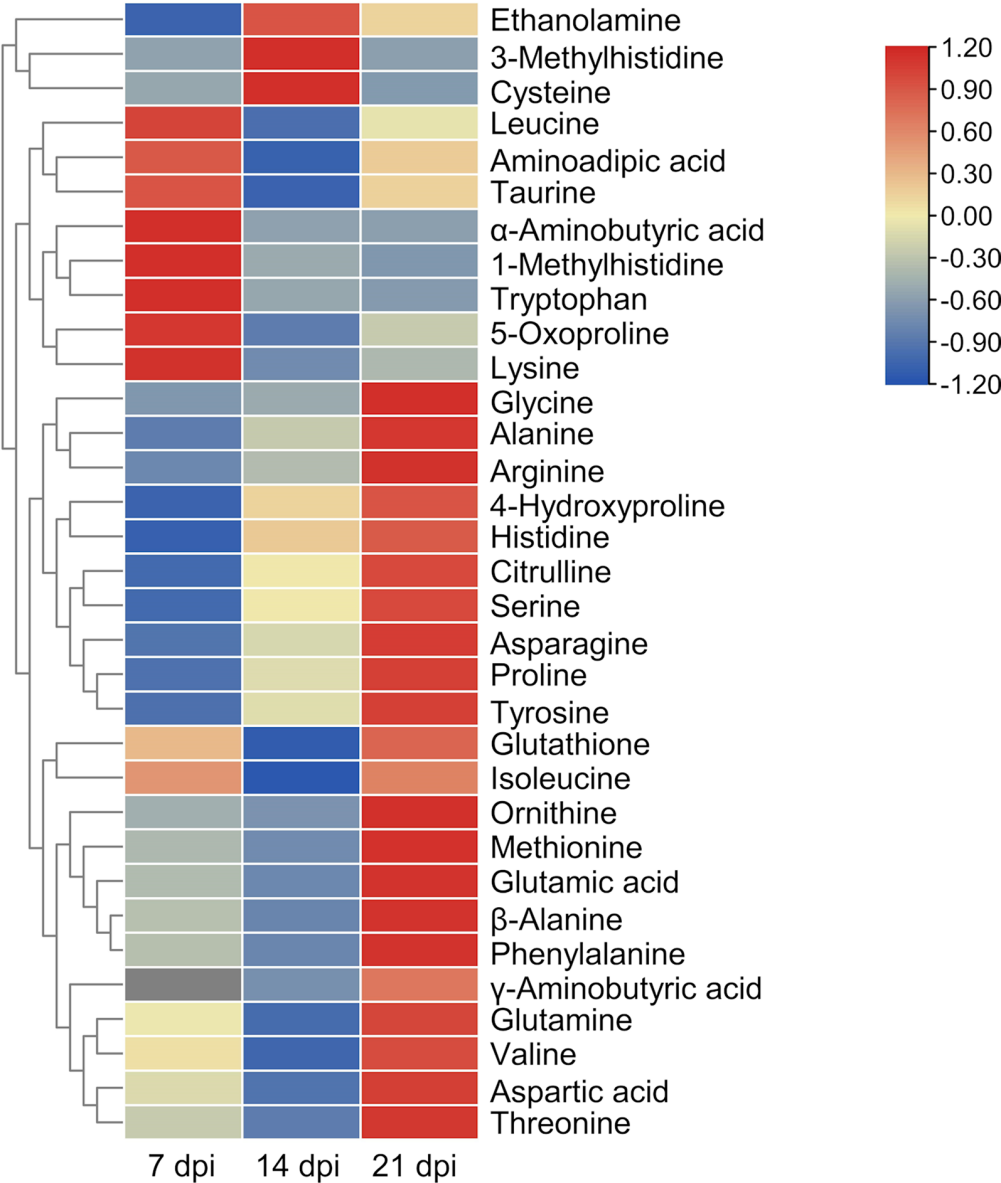
Heat map showing the profiles of 33 biogenic amino acids/AADs of control and IAV-infected mice. Each colored cell represents metabolite changes between the control and infection groups. The heat map was assembled by cluster analysis, and therefore, similar levels of metabolite changes are positioned at a short distance from each other, whereas different levels of metabolite changes are positioned at a long distance from each other. *n* = 5

**Fig. 2:**
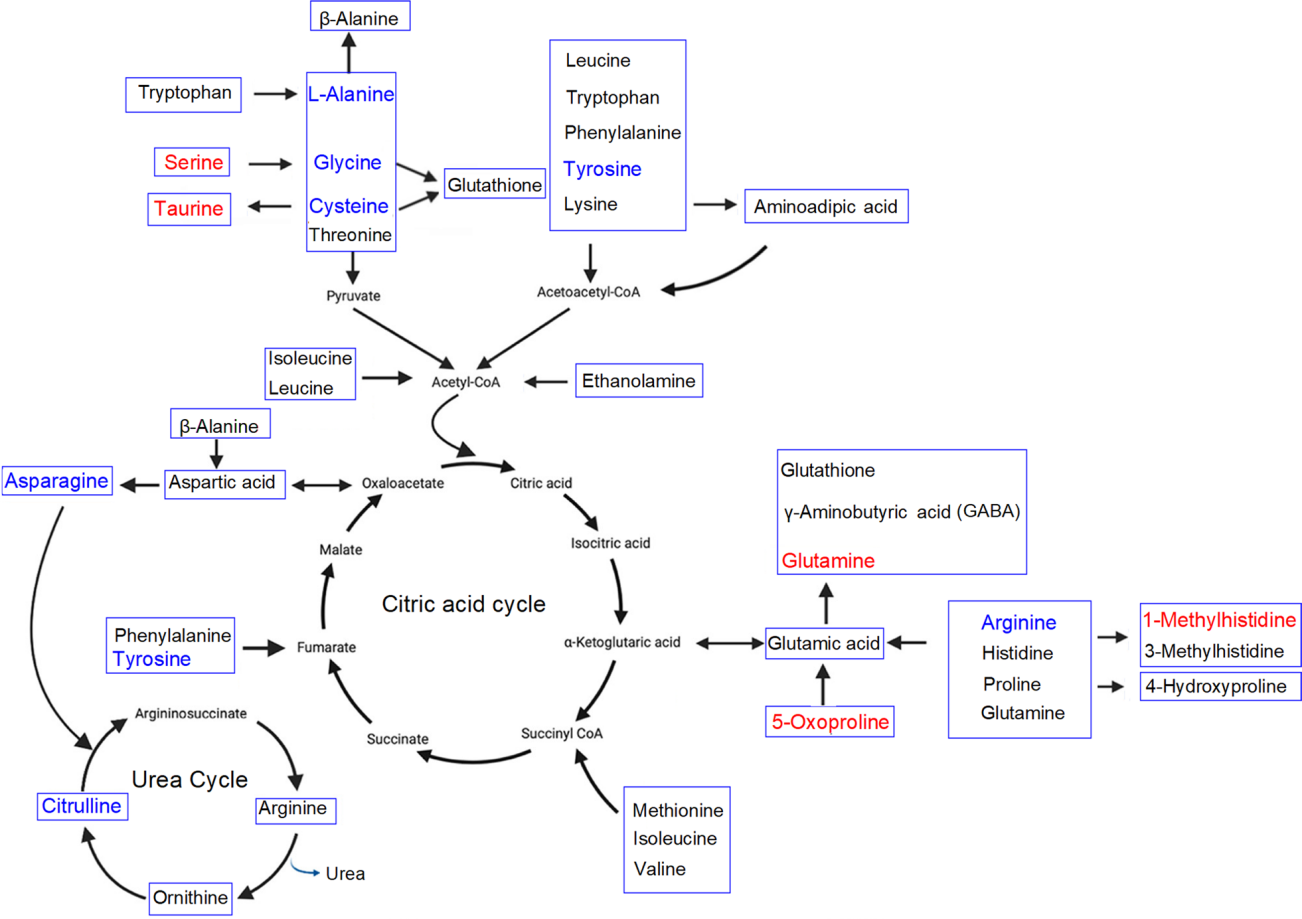
Metabolic pathway associations of significantly different biogenic amino acids/AADs at 7 dpi with IAV. Blue letters indicate a decrease in IAV infection, whereas red letters indicate an increase in IAV infection (*p* < 0.05)

The 33 amino acids/AADs profiles of control and IAV-infected mice during IAV infection (7 dpi, 14 dpi, and 21 dpi) are shown in Table 1 and Figs. 1 and 2. At 7 dpi, 32 amino acids/AADs were detected, but not γ-aminobutyric acid (GABA). Among the 32 amino acids/AADs, the levels of five amino acids/AADs (1-methylhistidine (*p* = 0.014), 5-oxoproline (*p* < 0.01), α-aminobutyric acid (*p* = 0.016), glutamine (*p* = 0.03), and taurine (*p* < 0.01)) increased, whereas the levels of ten amino acids/AADs (4-hydroxyproline (*p* = 0.026), alanine (*p* = 0.020), arginine (*p* = 0.026), asparagine (*p* = 0.029), cysteine ((*p* < 0.01), citrulline (*p* < 0.01), glycine (*p* = 0.01), methionine (*p* = 0.04), proline (*p* = 0.02), and tyrosine (p = 0.03)) decreased. No significant differences were observed in the levels of other amino acids/AADs between the control group and IAV-infected group.

**Table 1:** Univariate analyses of changes in the amino acid concentrations in blood plasma between control and infected mice at 7, 14, and 21 dpi

Amino acid	Changes in amino acid concentrations between control and infected mice					
	Change at 7 dpi (µM)	*p*	Change at 14 dpi (µM)	*p*	Change at 21 dpi (µM)	*p*
1-Methylhistidine	*5.98*	*0.014*	0.36	0.18	− 0.14	0.38
3-Methylhistidine	− 0.41	0.28	*0.58*	*0.03*	− 0.15	0.41
4-Hydroxyproline	− **6.69**	**0.026**	− 0.70	0.31	3.19	0.06
5-Oxoproline	*127.12*	< *0.01*	61.37	0.21	81.92	0.51
Alanine	− **466.63**	**0.02**	− 260.63	0.11	197.04	0.11
α-Aminobutyric acid	*12.72*	*0.016*	− **1.18**	**0.03**	− 1.32	0.10
Aminoadipic acid	3.02	0.14	− **4.26**	**0.01**	0.43	0.44
Arginine	− **41.81**	**0.026**	− 36.89	0.16	− 18.71	0.28
Asparagine	− **26.80**	**0.029**	− 7.98	0.22	21.73	0.09
Aspartic acid	− 2.94	0.14	− 42.08	0.23	53.67	0.08
β-Alanine	− 0.37	0.30	− 3.08	0.19	8.31	0.111
Citrulline	− **30.66**	** < 0.01**	− 2.92	0.42	23.74	0.09
Cysteine	− **24.76**	** < 0.01**	9.88	0.38	− 26.96	0.14
Ethanolamine	− 0.49	0.34	5.86	0.12	3.35	0.07
γ-Aminobutyric acid	ND	NA	− 0.26	0.26	0.43	0.18
Glutamic acid	− 12.71	0.06	− 42.77	0.24	95.66	0.11
Glutamine	*107.76*	*0.03*	21.94	0.39	*198.87*	*0.023*
Glutathione	− 1.99	0.32	− 29.19	0.12	*7.94*	*0.01*
Glycine	− **64.36**	**0.01**	− 47.44	0.24	*144.27*	*0.02*
Histidine	− 13.54	0.11	12.38	0.16	26.24	0.06
Isoleucine	− 6.03	0.25	− 23.66	0.05	− 4.86	0.34
Leucine	15.63	0.16	− 18.71	0.19	− 2.76	0.44
Lysine	37.40	0.26	− 34.39	0.26	− 20.58	0.36
Methionine	− **11.02**	**0.04**	− **14.32**	** < 0.01**	3.30	0.10
Ornithine	− 28.95	0.05	− 44.42	0.27	81.26	0.13
Phenylalanine	4.75	0.28	− 6.70	0.23	42.11	0.08
Proline	− **84.63**	**0.02**	− 27.52	0.21	50.40	0.17
Serine	− 91.98	0.06	− 12.90	0.32	61.95	0.05
Taurine	*1223.15*	< *0.01*	− 307.35	0.29	631.54	0.08
Threonine	− 42.72	0.07	− **77.87**	**0.01**	33.66	0.09
Tryptophan	1.19	0.46	− 3.77	0.39	− 4.08	0.33
Tyrosine	− **39.46**	**0.03**	− 6.24	0.37	38.29	0.11
Valine	− 10.56	0.09	− **45.37**	**0.02**	18.12	0.23

Bold numbers indicate a increase in IAV infection, whereas italic numbers indicate an decrease (p < 0.05) in IAV infection. *ND* Not detected, *NA* Not applicable

At 14 dpi, all 33 amino acids/AADs were detected; among these 33 amino acids/AADs, the levels of one AAD (3-methylhistidine (*p* = 0.03)) increased, whereas the levels of five amino acids/AADs (α-aminobutyric acid (*p* = 0.03), aminoadipic acid (*p* = 0.01), methionine (*p* < 0.01), threonine (*p* = 0.01), valine (*p* = 0.02)) decreased. No significant differences were found in the levels of other amino acids/AADs between the control group and IAV-infected group.

At 21 dpi, all 33 amino acids/AADs were detected. Among these 33 amino acids/AADs, only the levels of three amino acids/AADs (glutamine (*p* = 0.023), glutathione (*p* = 0.01), glycine (*p* < 0.01)) increased. No significant differences were detected in the levels of other metabolites between the control group and IAV-infected group.

### Changes in metabolites of tryptophan pathway during IAV infection

**Fig. 3:**
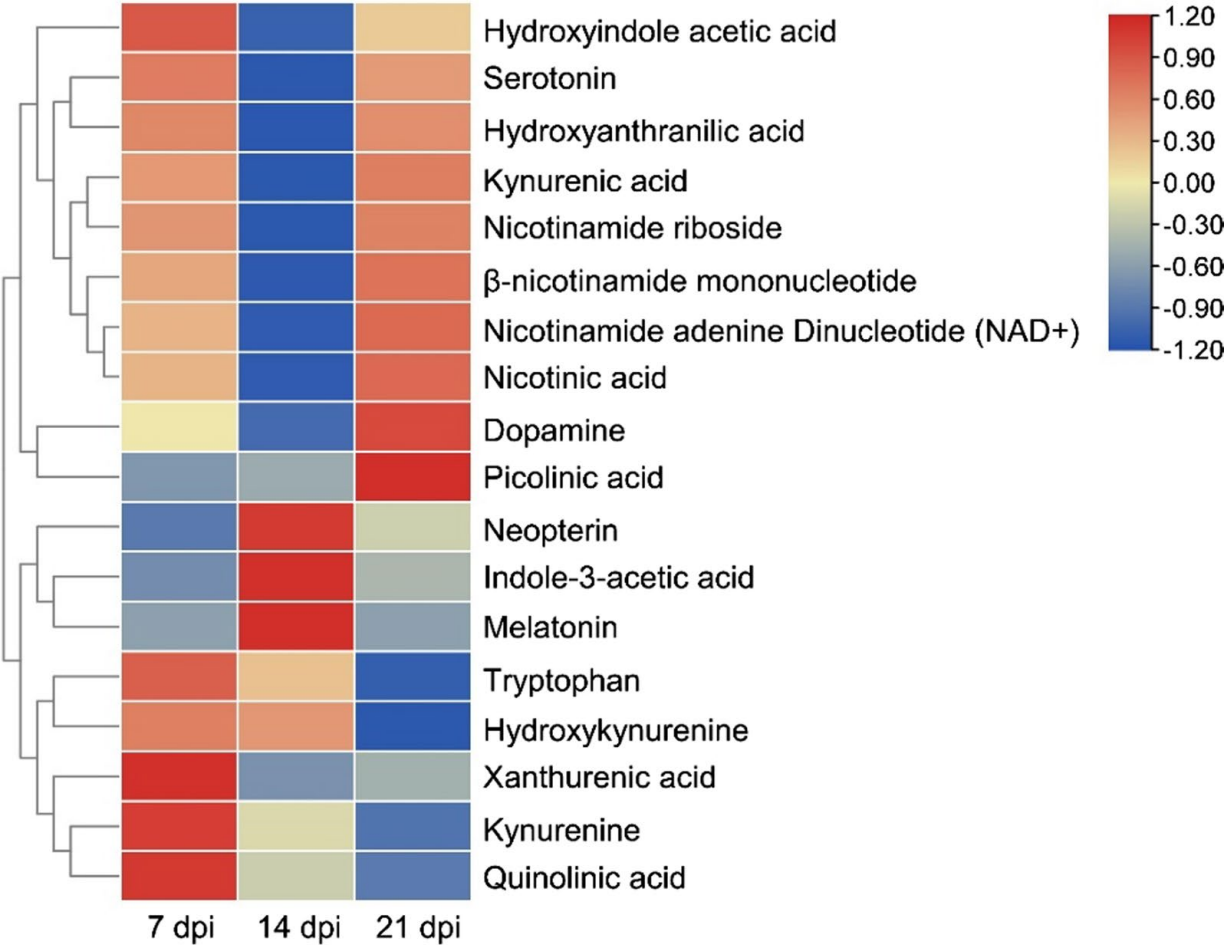
Heat map showing 18 metabolites of the tryptophan pathway during IAV infection. Each colored cell represents metabolite changes between the control and infection groups. The heat map was assembled by cluster analysis, and therefore, similar levels of metabolite changes are positioned at a short distance from each other, whereas different levels of metabolite changes are positioned at a long distance from each other. *n* = 5

**Fig. 4:**
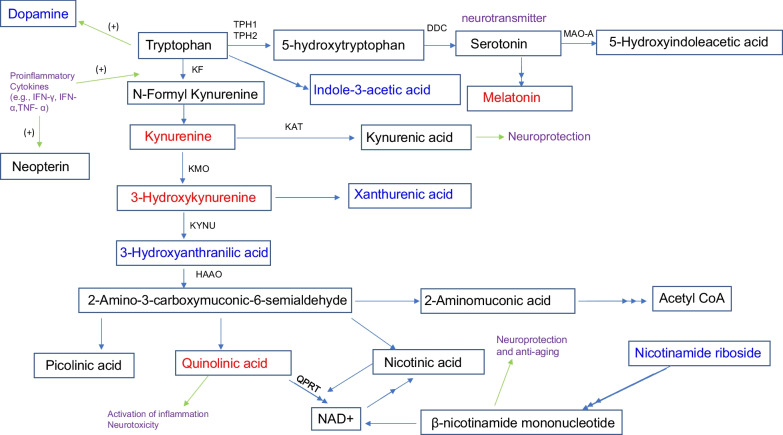
Metabolic pathway associations of significantly different metabolites of tryptophan pathway during IAV infection. Blue letters indicate a decrease in IAV infection, whereas red letters indicate an increase (*p* < 0.05) in IAV infection. *IFN-γ* Interferon-γ, *IFN-α* Interferon-α, *TNF-α* Tumor necrosis factor-α, *TPH1/2* Tryptophan hydroxylase 1/2, DDC, 3,4-dihydroxyphenylalanine decarboxylase, *MAO-A* Monoamine oxidase A/B, *IDO1/2* Indoleamine-2,3-dioxygenase 1/2, *TDO2* Trypto-phan2,3-dioxygenase, *KF* Kynurenine formamidase, *KAT* Kynurenine aminotransferase, *KMO* Kynurenine 3-monooxygenase, *KYNU* Kynurenine hydrolase, *HAAO* 3-hydroxyanthranilic acid dioxygenase, *QPRT* Quinolinic acid phosphoribosyl transferase

We then analyzed 18 plasma metabolites (including tryptophan) of the tryptophan pathway during IAV infection; the results are shown in Table 2 and Figs. 3 and 4. At 7 dpi, the levels of kynurenine (*p* < 0.01), quinolinic acid (*p* < 0.01), hydroxykynurenine (*p* < 0.01) increased, whereas the levels of indole-3-acetic acid (*p* < 0.01) and nicotinamide riboside (*p* < 0.01) decreased compared with the corresponding control groups. No significant differences were found in the levels of other metabolites between the control group and IAV-infected group. At 14 dpi, the levels of kynurenine (*p* < 0.01) and quinolinic acid (*p* = 0.04) increased, whereas the levels of dopamine (*p* = 0.04) and xanthurenic acid (*p* = 0.04) decreased compared with the corresponding control groups. No significant differences in the levels of other metabolites were seen between the control group and IAV-infected group. At 21 dpi, no significant differences were apparent between the levels of any of the metabolites when the control group and IAV-infected group were compared.

**Table 2:** Changes in levels of 18 metabolites of the tryptophan pathway between control and infected mice at the acute, resolution, and recovery stages of IAV infection

Metabolites of the tryptophan pathway	Concentration change at 7 dpi between control and infected group		Concentration change at 14 dpi between control and infected group		Concentration change at 21 dpi bewteen control and infected group	
	Change (µM)	*p*	Change (µM)	*p*	Change (µM)	*p*
β-nicotinamide mononucleotide	0.53	0.78	− 291.46	0.053	59.57	0.58
Dopamine	− 0.19	0.37	**− 2.10**	**0.028**	1.64	0.21
Indole-3-acetic acid	**− 290.80**	**0.0005**	− 84.31	0.46	− 255.57	0.16
Kynurenic acid	− 0.31	0.84	− 2.54	0.28	− 0.08	0.97
Kynurenine	*137.27*	*0.0086*	32.94	0.41	− 37.76	0.52
Melatonin	0.00	0.97	0.01	0.99	0.00	0.99
Neopterin	− 0.03	0.90	0.26	0.68	0.07	0.89
Nicotinamide adenine Dinucleotide (NAD^+^)	14.22	0.10	− 202.85	0.35	81.13	0.26
Nicotinamide riboside	− 2.56	0.67	−** 40.29**	**0.027**	0.03	0.99
Nicotinic acid	− 1.40	0.66	− 34.27	0.12	9.01	0.43
Picolinic acid	− 6.34	0.18	− 5.50	0.21	3.10	0.75
Quinolinic acid	23.59	0.39	− 28.43	0.64	− 54.32	0.53
Serotonin	1.05	0.21	− 2.44	0.07	0.68	0.79
Tryptophan	0.97	0.58	− 0.23	0.96	− 2.98	0.13
Hydroxyanthranilic acid	**− 0.76**	**0.036**	− 25.34	0.27	− 1.35	0.37
Hydroxykynurenine	3.70	0.036	2.94	0.40	− 5.68	0.35
Hydroxyindole acetic acid	− 12.56	0.60	− 147.48	0.32	− 60.23	0.34
Xanthurenic acid	0.56	0.61	**− 4.95**	**0.024**	− 4.25	0.47

Bold numbers indicate a decrease in IAV infection, whereas italic numbers indicate an increase (*p* < 0.05) in IAV infection

## Discussion

**Fig. 5:**
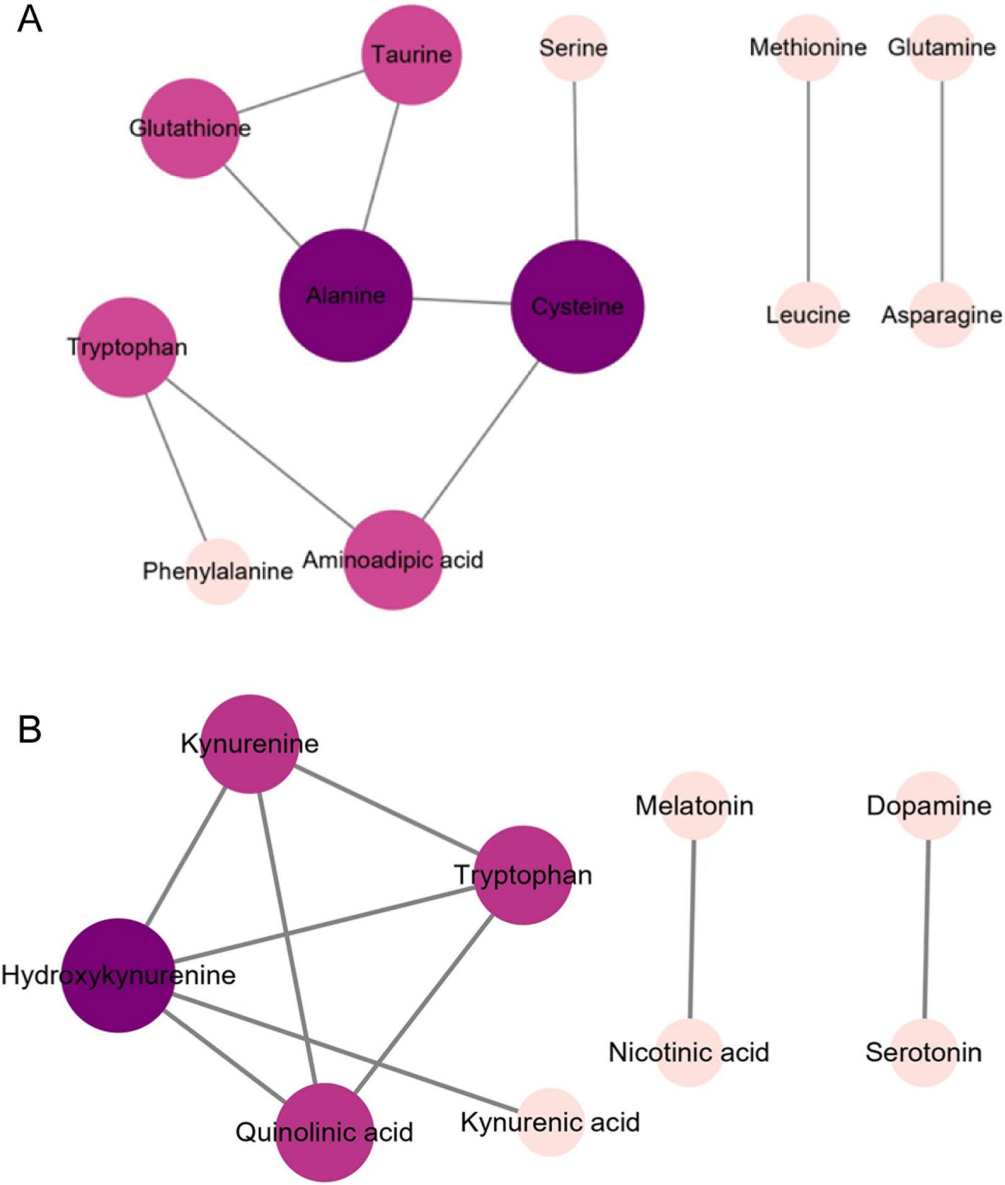
Metabolic correlation network diagram for metabolites analyzed during IAV infection. **A** Metabolic correlation network for biogenic amino acids/AADs analyzed during infection. **B** Metabolic correlation network for metabolites of the tryptophan pathway analyzed during infection. Each connected node represents structurally related metabolites, as determined by molecular networking. The metabolic data were imported into the Cytoscape software to form a differential metabolites network of interaction. The larger the area of the circle, the darker the color and the higher the degree values

Immunometabolism is the molecular and biochemical study of the metabolic regulation of immune function and immune regulation of the metabolism system [42]. We have investigated the effect of IAV infection on the blood plasma levels of 51 metabolites during the acute, resolution, and recovery stages of virus infection. Changes in the metabolites and associated metabolic pathways during the infection are summarized in Figs. 1 and 3. The metabolic correlations between the investigated metabolites are presented in Fig. 5.

Among these metabolites, several amino acids are neurotransmitters; their changes during the infection are summarized in Table 3. Influenza viruses can be classified into two main categories based on their ability to infect and affect the nervous system: neurotropic and non-neurotropic [43–47]. The vast majority of influenza infections are caused by non-neurotropic influenza viruses. Like many other internal organs, the lung is innervated by sympathetic, parasympathetic, and sensory nervous systems that regulate the function of cells within the respiratory tract [48–51]. The neuroimmune interactions in the lung might have essential roles in the host′s anti-viral immune response [49, 52]. The H1N1 virus used in our study is a non-neurotropic influenza virus [46, 53].

**Table 3:** Changes in levels of 8 neurotransmitters between control and infected mice at the acute (7 dpi), resolution (14 dpi), and recovery stages (21 dpi) of IAV infection

Amino acids/ neurotransmitters	Excitatory or inhibitory	Changes during infection
Aspartic acid	Excitatory	No significant difference
β-alanine	Inhibitory	No significant difference
Cysteine	Excitatory	Decrease at 7 dpi
GABA	Inhibitory (adult); excitatory (developing)	No significant difference
Glycine	Inhibitory	Decrease at 7 dpi Increase at 21 dpi
Glutamic acid	Excitatory	No significant difference
Taurine	Inhibitory	Increase at 7 dpi
Glycine	Inhibitory	Decrease at 7 dpi Increase at 21 dpi
Dopamine	Both excitatory and inhibitory	Decrease at 14 dpi
Serotonin	Inhibitory	No significant difference

Unsurprisingly, the major changes occur within 7 dpi agreeing with the clinical spectrum of IAV infection in which signs and symptoms of uncomplicated influenza typically resolve after 3–7 days for most patients [3].

### IAV infection increases levels of some biogenic amino acids/AADs

1-Methylhistidine is a histidine derivative with a methyl group bound to the nitrogen at position 1 and results from the metabolism of dipeptide anserine obtained from food [54]. We have observed an increased level of 1-methylhistidine at 7 dpi. Elevated levels of 1-methylhistidine may indicate an increased uptake of short-chain peptides in the food, a possible increase in gut permeability, decreased digestive peptidase activity, and incomplete digestion in the small intestine [54]. In addition, we have observed an elevated level of 3-methylhistidine, which is also a histidine derivative, at 14 dpi. Formed by the post-translational methylation of histidine residues in the myofibrillar proteins (actin and myosin), 3-methylhistidine is released after the proteolysis of the two proteins [55]. The increased level of 3-methylhistidine may indicate muscle protein breakdown (muscle wasting) during the infection [55].

Taurine, an abundant essential amino acid, has important functions in numerous biological pathways (e.g., homeostasis, anti-oxidation, calcium signaling, bile-salt formation, anti-inflammation, membrane stabilization, and osmoregulation) [56–58]. It also regulates various cellular processes including energy metabolism, gene expression, the quality control of proteins, and neuroprotection [59]. Previous studies have shown that the level of taurine is upregulated during human SARS-CoV-2 infection [15].Taurine probably protects the liver from damage/injury and improves lipid profiles [60]. Therefore, we were not surprised to find that, at the acute stage, our IAV-infected mice had significantly higher level of taurine. Our results concerning taurine are also consistent with a previous study showing elevated taurine level at 8 dpi with IAV [10]. The elevation of plasma taurine is an indication of liver dysfunction or damage [15, 61].

Glutamine, which is a primary energy-providing substrate of immune cells, plays a vital role in their function, homeostasis, and proliferation by being an essential precursor for the synthesis of purine and pyrimidine nucleotides [62]. Glutamine deficiency in high-risk patients suffering with COVID-19 has recently been identified as a predisposing factor in the development of the severity of the viral disease [63]. Glutamine transport/metabolism can be used as targets to develop anti-viral drugs [64]. Glutamine is also pivotal in the proper functioning of the nervous system since it is important in the production of both excitatory (glutamate) and inhibitory (GABA) neurotransmitters in the brain [65]. An insufficient supply of glutamine to neurons might be the cause of depressive behaviors [66]. In our work, we have observed that glutamine levels are elevated at 7 dpi and 21 dpi indicating a potential effect of infection not only on immunity, but also on the nervous system [15]. We did not detect GABA at 7 dpi since its amount might be very little and below our detection limit.

5-Oxoproline is a ubiquitous (but little studied) natural amino acid derivative in which the free amino group of glutamic acid or glutamine cyclizes to form a lactam [67]. In the present study, we have detected a higher level of 5-oxoproline at 7 dpi. Accumulation of 5-oxoproline can occur in the blood, possibly leading to high anion gap metabolic acidosis, especially after the use of drugs such as paracetamol, flucloxacillin, and vigabatrin [68]. Since acidosis can regulate cellular responses by affecting enzyme activity, ion transport, protein/DNA synthesis, and the levels of 3',5'-cyclic adenosine monophosphate (cAMP)/calcium, an acidic microenvironment might impair immune cell functions [69]. In addition, acidosis can also impair brain functions because of damage to neurons/glial cells and an imbalance between the cortical pyramidal neurons and GABAergic neurons (a process similar to neural excitotoxicity) [70]. Since 5-oxoproline can be converted back into L-glutamate by 5-oxoprolinase, it might have essential roles in glutamate storage and oppose the action of glutamate in the brain. In our study, the increase of 5-oxoproline might be attributable to infection-stimulated malnutrition.

We observed a sharp increase of α-aminobutyric acid at 7 dpi and a slight decrease at 14 dpi. α-Aminobutyric acid is a non-essential amino acid derived from methionine, threonine, and serine. It has been linked with various conditions such as alcoholic liver injury, infection/sepsis, malnutrition, and multiple organ failure [71]. In addition, the elevation might indicate inadequate utilization of this amino acid for cellular energy generation, in which it is converted to succinyl CoA for use in the citric acid cycle via mechanisms requiring biotin and Vitamin B12 [71].

Glycine is a non-essential amino acid under steady conditions, as are glutamine, proline, and serine, but it becomes essential during diseases and in stress states. It plays a vital role in metabolic regulation, anti-oxidative reactions, infection/inflammation, and neurological functions [72]. In the present study, the level of glycine decreased at 7 dpi, whereas its level increased at 21 dpi.

The importance of glycine is well known in the maintenance of metabolic health [73]. A low glycine level in blood plasma is associated with metabolic disorders, including non-alcoholic fatty liver disease, type-2 diabetes, and obesity, and is a risk factor for the severity of influenza [74]. Moreover, as an inhibitory neurotransmitter, it contributes to the processing of the motor and sensory information that permits movement, vision, and audition [72]. A low level of glycine is associated with poor feeding, lack of energy, weak muscle tone, breathing problems, seizures, and coma. The blocking of glycine receptors has also been reported to reduce neuroinflammation and to restore neurotransmission in the central nervous system (CNS) [75]. In addition, glycine has essential functions in the immune response to/inflammation during infection. For example, in the early stage of COVID-19, glycine might suppress the onset of virus-induced cytokine storm. In the late stages of the disease, it might protect lung tissues from severe damage and acute respiratory distress syndrome which are two leading causes of mortality [75]*.* Glycine intake (or a higher level of glycine) might prevent and help in the fight against virus infection by strengthening the extracellular matrix [76].

### IAV infection decreases levels of some biogenic amino acids/AADs

Alanine is a non-essential amino acid involved in sugar metabolism (e.g., in muscles and CNS) and immune system functions, specifically T lymphocyte activation [77, 78]. Alanine can bind to the glycine site of N-methyl-d-aspartate (NMDA) receptors in the brain and improves the positive and cognitive symptoms of patients with schizophrenia [79]. In this study, we have observed a sharp decrease in alanine at 7 dpi. However, we have not found a meaningful change in β-alanine during the infection. This lower alanine level might be attributable to inadequate protein intake, low levels of branched-chain amino acids (valine, leucine, and isoleucine), gastrointestinal malabsorption/maldigestion, and/or increased demands in gluconeogenesis.

Recent studies have shown that the levels of many amino acids (e.g., valine, proline, citrulline, isoleucine, asparagine, and arginine) and their derivatives can change after SARS-CoV-2 infection, and that these changes are associated with disease severity [80]. A decrease in amino acids might be related to inadequate protein intake, and malabsorption/maldigestion as observed in the above-mentioned decreased level of alanine.

Arginine is an essential nutrient for both the innate and the adaptive immune systems [81]. It is also a precursor for nitric oxide (NO), which is a powerful neurotransmitter that helps blood vessels relax and improves circulation [15]. NO might also inhibit virus replication and be used to restore T cell function [15]. For example, arginine might inactivate enveloped viruses (e.g., IAV) at an acidic pH (or elevated temperature) and can be used as a disinfectant and treatment of the viral infection [82]. Therefore, a deficiency in arginine might contribute to the pathogenesis of viral diseases.

Asparagine is a non-essential amino acid that might have a metabolism-independent role in regulating the adaptive immune response by controlling T-cell activation and efficacy [83]. The decrease of asparagine observed in our study probably limits the replication of virus, as reported in a previous study [84].

L-citrulline has recently been suggested to prevent neuronal death and to protect against cerebrovascular injury; the supply of L-citrulline to the brain is a possible treatment for the improvement of its neuroprotective effect in patients with cerebrovascular disease [85]. L-citrulline together with arginine has been linked to the ability to boost the immune system and help fight pathogens naturally [86]. For example, one study has shown that T cells might rely on L-citrulline in microenvironments lacking L-arginine to maintain proliferation and cytokine production [87].

Cysteine is a non-essential sulfur-containing amino acid. It can inhibit oxidative stress, which in turn maintains immune system function and health [88]. Cysteine can also function as an excitatory neurotransmitter [89]. Proline, a non-essential amino acid, has essential roles in protein (e.g., collagen) synthesis/ structure, metabolism (especially the synthesis of arginine, polyamines, and glutamate), wound healing, anti-oxidative reactions, and immune responses [90]. Tyrosine is a non-essential amino acid made from phenylalanine. It is the precursor of several neurotransmitters such as epinephrine, norepinephrine, and dopamine. It is used as a supplement to improve alertness, attention, and focus. Depending on its dose, it might boost both physical and mental performance [91].

### IAV infection alters the levels of some metabolites in the tryptophan pathway

Kynurenine is a tryptophan metabolite produced by indoleamine 2,3-dioxygenase (IDO1)/tryptophan-2,3-dioxygenase-2 (TDO-2; Fig. 4), and the kynurenine pathway is responsible for metabolizing most of the free tryptophan in animals. This pathway can be activated by infectious agents, inflammatory mediators, and stress. In the past decades, the kynurenine pathway has received increasing attention because of its association with inflammation, the immune response, and neurological disorders/conditions [15, 92, 93]. Dysregulation or overactivation of the kynurenine pathway causes immune system activation and the accumulation of potentially neurotoxic compounds (e.g., quinolinic acid and 3- hydroxykynurenine) [15]. The increased level of kynurenine (7 dpi and 14 dpi) observed in our study agrees with a previous report showing an activated kynurenine pathway by using mouse models of IAV infection [94]. In addition, the increased level of kynurenine might contribute to the production of kynurenic acid, which has a neuroprotection role [15].

In the current study, we have observed a sharp decrease in indole-3-acetic acid at the acute stage of IAV infection. indole-3-acetic acid is a breakdown product of tryptophan metabolism and is often produced by the bacteria in the mammalian gut [95]. Recent studies have shown that indole-3-acetic acid has anti-inflammatory and anti-oxidative activities [96]. For example, it can modulate intestinal homeostasis and suppress inflammatory responses. In addition, IAA has previously been linked to SARS-CoV-2 infections [15]. Furthermore, indole-3-acetic acid can affect neurological functions. For example, it has recently been demonstrated to have anti-depressive effects under certain conditions [97].

Dopamine is a monoamine neurotransmitter associated with movement, attention, learning, and the brain's pleasure/reward system. Phenylalanine and tyrosine constitute the two initial steps in the synthesis of dopamine, which is the metabolic precursor of epinephrine and norepinephrine [98]. In our study, we have observed a decrease in dopamine at 14 dpi. Dopamine has recently been linked with viral infection and the host immune response. For example, dopamine and serotonin participate in the pathophysiology of COVID-19 [99]. IAV (H1N1) also has a high affinity to dopaminergic neurons in the CNS [100]. In addition, many immune cells express dopamine receptors, and dopamine can modulate immune cell functions after binding with dopamine receptors.

## Conclusions

Our blood plasma metabolite profiling has revealed correlations of specific metabolites with infection by IAV. The results may improve our understanding of the molecular mechanisms of host responses to such infections and provide insights for producing potential novel mechanistic, diagnostic, and prognostic biomarkers (including those associated with neuro-immune interactions) and of therapeutic targets. In particular, although the significant imbalance between those metabolites that are associated with neurotoxicity (e.g., quinolinic acid, 3-hydroxykynurenine, and serine), those that are neuroprotective (kynurenic acid), and those that are neurotransmitters (e.g., glutamic acid, glycine, and dopamine) observed following IAV infection in this study needs further investigation, our findings may elucidate the mechanism by which IAV causes acute and long-term primary/secondary neuroinflammatory/neurological disorders/complications (e.g., seizures, encephalopathy, brain fog, encephalitis, stroke, focal neurologic deficits, Guillain-Barré syndrome, acute disseminated encephalomyelitis, and transverse myelitis) [44]. Further studies are needed to determine whether a specific unique phenotype of IAV infection occurs, or whether the phenotype is a result of the overall systemic impact/reaction of the infectious disease.

### Supplementary Information

**Additional file 1 Fig. S1** Body weight changes of mice 3 dpi, 7 dpi, 14 dpi, and 21 dpi after H1N1 virus infection.

## Data Availability

The datasets supporting the conclusions of this article are included within the article and its additional files.

## Abbreviations

IAV: Influenza A virus
AADs: Amino acid derivatives
UHPLC-ESI–MS/MS: Ultra-High-Performance Liquid Chromatography-Mass Spectrometry with Electrospray Ionization
dpi: Days post-infection
SARS-CoV-2: Severe acute respiratory syndrome coronavirus 2
COVID-19: Coronavirus disease of 2019
HA: Hemagglutinin
MDCK: Madin-Darby canine kidney
DMEM: Dulbecco´s modified Eagle′s medium
TCID 50: Tissue culture infective dose that killed 50% of the cells
UHPLC-MS: Ultra-High-Performance Liquid Chromatography-Mass Spectrometry
SIL: Stable isotope-labeled
LC–MS: Liquid Chromatography-Mass Spectrometry
QToF: Quadrupole time-of-flight
ESI: Electrospray ionization
MS: Mass spectrometry
MS/MS: Tandem Mass Spectrometry
bbCID: Broadband collision-induced dissociation
TASQ: Target Analysis for Screening and Quantification
GABA: γ-Aminobutyric acid
cAMP: 3',5'-Cyclic adenosine monophosphate
CNS: Central nervous system
NMDA: N-methyl-d-aspartate
NO: Nitric oxide
IDO1: Indoleamine 2,3-dioxygenase
TDO-2: Tryptophan-2,3-dioxygenase-2
IFN-γ: Interferon-γ
IFN-α: Interferon-α
TNF-α: Tumor necrosis factor-α
TPH1/2: Tryptophan hydroxylase 1/2
DDC: 3,4-Dihydroxyphenylalanine decarboxylase
MAO-A: Monoamine oxidase A
IDO1/2: Indoleamine-2,3-dioxygenase 1/2
KF: Kynurenine formamidase
KAT: Kynurenine aminotransferase
KMO: Kynurenine 3-monooxygenase
KYNU: Kynurenine hydrolase
HAAO: 3-Hydroxyanthranilic acid dioxygenase
QPRT: Quinolinic acid phosphoribosyl transferase
NAD: Nicotinamide adenine dinucleotide
