# Supplementary figures and images for: Systemic perturbations in amino acids/amino acid derivatives and tryptophan pathway metabolites associated with murine influenza A virus infection
Source: Virol J. 2023 Nov 21;20:270. doi: 10.1186/s12985-023-02239-0 (PMC10664681; doi:10.1186/s12985-023-02239-0)

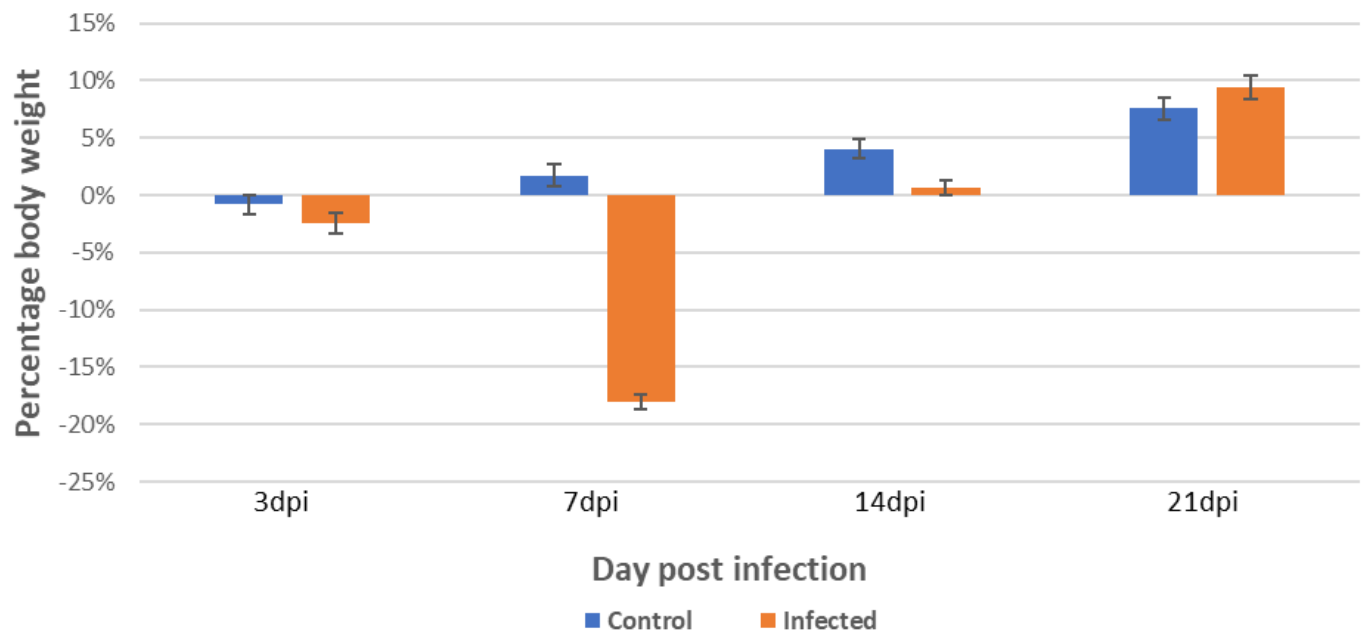

Supplementary Figure 1 Body weight changes of mice 3 dpi, 7 dpi, 14 dpi, and 21 dpi after H1N1 virus infection.

Supplement: Supplementary file 1 — Additional file 1 Fig. S1 Body weight changes of mice 3 dpi, 7 dpi, 14 dpi, and 21 dpi after H1N1 virus infection. [file 12985_2023_2239_MOESM1_ESM.pdf]
